# Slow stamen movement in a perennial herb decreases male–male and male–female interference

**DOI:** 10.1093/aobpla/plx018

**Published:** 2017-06-07

**Authors:** Lingyan Wang, Yu Bao, Hanxi Wang, Chunguang He, Ping Wang, Lianxi Sheng, Zhanhui Tang

**Affiliations:** a State Environmental Protection Key Laboratory of Wetland Ecology and Vegetation Restoration, School of Environment, Northeast Normal University, Jingyue Street 2555, Changchun 130024, China

**Keywords:** Adaptive evolution, dichogamy, herkogamy, male**–**female interference, male**–**male interference, pollen dispersal mechanism, slow stamen movement

## Abstract

Approximately 80 % of angiosperm species produce hermaphroditic flowers, which face the problem of male**–**male sexual interference (one or more anthers gets in the way of disseminating pollen from other anthers) or male**–**female sexual interference (the pistil interferes with disseminating pollen from the anthers by preventing the anther from touching a pollinator, or the anther prevents pollinator from depositing outcross pollen on the stigma). Slow stamen movement in hermaphrodite flowers has been interpreted as an adaptation for reducing male**–**male sexual interference. Using slow stamen movement in *Lychnis cognata* (Caryophyllaceae), this study presents new evidence that this phenomenon can reduce both male**–**male and male**–**female sexual interference. Ten stamens in *L. cognata* flowers vertically elongated their filaments in two batches and displayed similar patterns in pollen dispensing. More importantly, 10 stamens bend out of the floral centre by curving the filament also in 2 batches and pollen grains located at the flower centre displayed the highest viability. Thus, three stages of stamen movement can be identified, comprising two male stages (M1 and M2) and one female stage (F). We found that the main pollinator for *L. cognata, Bhutanitis yulongensis* (Papilionodae) generally preferred M1 flowers. Manipulation experiments show that vertical stamen movement enabled the anthers to dehisce at different times to prolong the presentation of pollen grains. Horizontal movement of the stamen decreased both male**–**male and male**–**female interference. However, vertical stamen movement had a minor role in increasing amount of pollen received by the stigma. This study provides the first direct experimental evidence of concurrent male**–**male and male**–**female interference in a flower. We suggest that the selection pressure to reduce such interference might be a strong force in floral evolution. We also propose that other selective pressure, including pollen dispensing mechanisms, pollen longevity, pollinator behaviour and weather, might contribute to floral evolution.

## Teaser

This study suggested that slow stamen movement in Lychnis cognata presents pollen gradually to pollinators, representing an adaptation to decrease interference between dehisced and dehiscing anthers and pistils that could promote pollen export and deposits. Adaptations that decrease both male-male sexual interference and male-female sexual interference should be recognized as important selective forces in floral evolution. This study also proposed that other selective pressures, including pollen dispensing mechanisms, pollen longevity, pollinator behavior and weather, might contribute to floral evolution.

## Introduction

Stamen movement refers to situations in which stamens move under their own volition or when they move in response to stimulation by external triggers such as pollinators (Ren 2010). Four main types of stamen movement are recognized (Ren 2010): stimulated (Cota-Sánchez *et al.* 2013); simultaneous and slow (Liu *et al.* 2006); quick and explosive (Joan *et al.* 2005); and cascade (Ren and Tang 2012). Slow stamen movement is widespread and is characterized by changes in stamen position, the degree of dichogamy during flowering, and the movement of the anther to a specific area that allows it to directly deliver pollen to recipient stigmas for self-fertilization (Ren 2010).

Slow stamen movement has been shown in *Idiospermum australiense* (family Calycanthaceae) (Lanteri *et al.* 2006; Du *et al.* 2012) and in the Orchidaceae (Webb and Lloyd 1986; Li *et al.* 2010). Over the 10–16-day floral lifespan of *Idiospermum australiense*, movement of floral organs causes spatial and temporal separation of male and female floral functions (Worboys and Jackes 2005). Similarly, stamen movement has been described to be responsible for the self-fertilization mechanism in the tree-living orchid, *Holcoglossum amesianum*, where the bisexual flower turns its anther 360°, against gravity, to insert pollen into its own stigma cavity, without the aid of any pollinating agent or medium (Liu *et al.* 2006).

Ren and Tang (2012) proposed that stamen movement can determine the fate of pollen. Percival (1955) first deciphered pollen dispensing mechanisms, and proposed the ‘pollen presentation theory’, which suggests that for a given number of pollinator visits, there will be an optimal chance for a plant to present pollen to the pollinators (Thomson *et al.* 2000). According to the ‘pollen presentation theory’, stamen movement can be regarded as a form of pollen ‘packaging’ and ‘dispensing’ that results in anthers dehiscing at different times to prolong the presentation of pollen grains. Recently, researchers have paid more attention to the phenomenon of ‘male**–**male’ interference, i.e. when one set of anthers gets in the way of disseminating pollen from another set of anthers. Ren and Bu (2014) provided direct experimental evidence for ‘anther–anther interference’ in angiosperms and showed its role in driving the evolution of floral traits, which had previously been ascribed to pollen dispensing mechanisms. However, the effects of male**–**female interference, i.e. the pistil interferes with disseminating pollen from the anthers by preventing the anther from touching a pollinator, or perhaps the anther prevents pollinator from depositing outcross pollen on the stigma, have been less well studied. It is likely that male**–**female interference may act as a selective force and needs to be considered when interpreting floral adaptation and evolution (Ren and Bu 2014). As stamen movements tend to result in movement herkogamy, they may decrease male**–**female interference by decreasing self-fertilization and increasing outcrossing (Webb and Lloyd 1986). Therefore, in most cases, slow stamen movement has an important influence on both pollen export and reception (Ren and Bu 2014). Indeed, slow stamen movement, along with herkogamy or dichogamy, alters the overall pattern of reproduction.

In a previous field study on *Lychnis cognata*, we observed slow stamen movement that involved a ‘five-by-five’ process: five stamens elongated their filaments on the first day and then curved their filaments on the second day after flowering. Subsequently, another five stamens repeated the same pattern the next day (unpublished observations). This system offers an ideal subject for studying the function of slow stamen movement and its importance as a selective force in floral adaptation and evolution. We tested the following hypotheses: (i) stamen vertical movement is a special type of ‘pollen dispensing mechanism’; (ii) stamen horizontal movement could decrease both male**–**male interference and male**–**female sexual interference, and then both pollen export and reception could be considered as important selective forces for floral adaptation and evolution in *L. cognata*.

## Methods

### Study site

The work was carried out in the Longwan National Nature Reserve (42°20′55.0″N, 126°20′12.5″E), which is located at an elevation of ∼741 m above mean sea level. It is situated on the western edge of the Longgang Mountain region, which is part of the Changbai Mountains in Northeast China. The annual precipitation in this region averages 704.2 mm and the mean temperatures in January and July are–15.7 and 22.5 °C, respectively. The vegetation type is classified as Changbai Mountain flora; the original vegetation, which mainly comprised *Pinus koraiensis* and broad-leaved mixed forests, has degraded into a secondary broad-leaved forest (Wang *et al.* 2017).

### Study species

**Figure 1.**
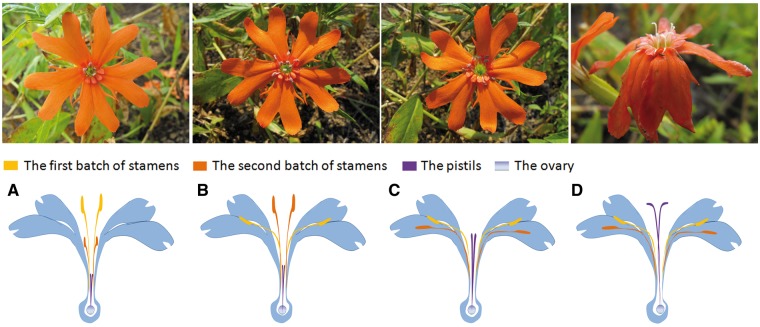
Photographs and diagrams of *L. cognata* flowers show the positions of stamens and pistils at different stages after flowering. (A) First day after flowering (M1 stage), the first batch of stamens occupy the optimal pollination position. (B) Second day after flowering (M2 stage), the second batch of stamens occupy the optimal pollination position. (C) Fourth day after flowering (F stage), pistils occupy the optimal pollination position and are outside of the tubular corolla. (D) Seventh day after flowering, the flowers are withered.

*Lychnis* *cognata* is a perennial herb native to most areas of northern China. It grows at the forest edge and in shrub grassland at altitudes between 500 and 1000 m above mean sea level. The plant grows to a height of 35–90 cm. It has dichotomous cymes with several orange-red or reddish flowers that contain 10 stamens and 5 pistils (Fig. 1); the flowers also contain a cylindrical, rod-shaped calyx, ovoid ovaries and oblong-ovate capsules. In the study area, *L. cognata* flower in July and fruits in August. The experiment was conducted on *L. cognata* population at the forest edge between July and September 2015.

### Vertical and horizontal stamen movement

To examine vertical stamen movement, we measured the lengths of stamens and pistils with an electronic vernier caliper (accurate to 0.001 cm); measurements were made at 0, 24, 48, 72 and 96 h after flowering from 10 to 21 August 2015. Twenty flowers were sampled at each time point, one flower per individual. 

**Figure 2.**
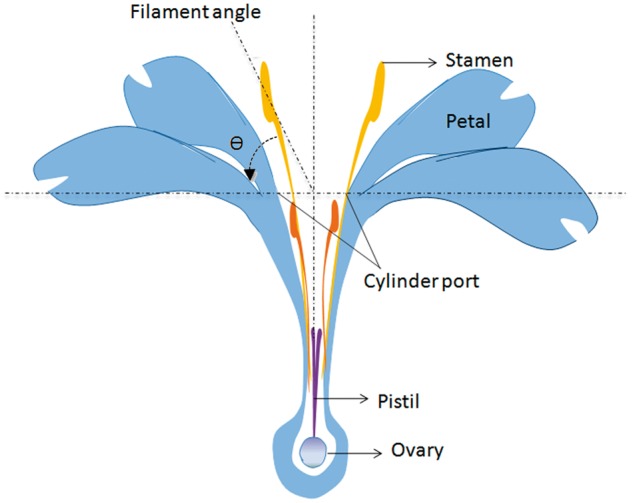
Filament angles in *L. cognata* flowers.

To examine horizontal stamen movement, we used a protractor to measure the angle of the filaments from the horizontal (Fig. 2) of the first five and second five of stamens at 0, 24, 48, 72 and 96 h, and 0, 10, 24, 34 and 48 h after flowering (10–21 August 2015). Five flowers were assessed for this endpoint, one flower per individual.

We chose flowers from plants of similar heights and of similar developmental stages to prevent potential influences from unidentified abiotic and biotic factors on the pattern of stamen movement.

### Pollen and stigma viability

During the inflorescence period 10–21 August 2015, 20 new flowers of *L. cognata* were randomly selected, one flower per individual. The flowers were bagged and marked each morning before flowering to obtain flowers unvisited by pollinators. Pollen from the first batch of stamens was collected at 0, 2, 4, 6, 8, 10, 24, 36, 48, 72 and 96 h after flowering; pollen from the second batch was collected at 24, 26, 28, 30, 32, 34, 48, 72, 96 and 120 h after flowering. The pollen samples were placed into a 1.5-mL centrifuge tube immediately following collection, and two drops of 1,2,3-triphenyl tetrazolium chloride were added to the tube with vigorous mixing. The pollen mixture was incubated at 35 °C for ∼20 min and then transferred onto a microscope slide and covered with a coverslip; pollen viability was observed with an Olympus Vanox microscope for active pollen that stained red. For each slide, red and unstained pollen grains were counted in three fields of vision at 100× magnification. Pollen viability was calculated as:

$$\text{Pollen viability}\;\text{=}\;\frac{\text{Red pollen grains}}{\text{red pollen grains}\;\text{+}\;\text{undyed pollen grains}}$$

We tested stigma viability in the field from 21 August to 18 September 2015. A total of 100 flowers were selected, 1 flower per individual. The flowers were manually pollinated at 0, 24, 48, 96 and 120 h (*n* = 20 flowers per treatment) after flowering. Assessment of the fruit-set rate was carried out at the end of the experiment.

### Pollinator preference for different flowering stages

Pollinator visitation behaviour was observed using binoculars from a distance of ∼10 m to avoid disturbance behaviour; these observations were made from 0700 to 1700 h on 6–8 August 2015, with a total of 30 h of observation. We did not conduct any nocturnal observations because pollinator visits to flowers were uncommon during the night (Wang, personal observation).

To determine pollinator preference at different flowering stages, we labelled a total of 49 flowers and recorded their flowering dates, one flower per individual. The visit duration of every pollinator at each flower was used to calculate the total visitation rate and visitation duration for each flowering stage. The flowering stage was defined by the stamen movement. As the first bench of stamens movement occurred during 0–24 h and the second batch of stamens movement occurred during 24–48 h after flowering, three stages of stamen movements were identified, which consisted of comprising two male stages after flowering (0–24 and 24–48 h after flowering, abbreviated as M1 and M2) and one female stage (48 h after flowering, abbreviated as F).

### Pollinator preference for different stamen movement patterns

A manipulation experiment was performed to interfere with stamen movement. For this experiment, 30 new flowers (15 assigned as controls, and 15 for treated) were randomly selected on 9 August 2015, 1 flower per individual. For the manipulation treatment, when the second batch of stamens uplifted their anthers slowly above the flower at 24 h after flowering, horizontal movement was stopped by fixing the first batch of stamens to the second batch at the top of filaments with a white thread. White threads were used because the filament was white, and a white thread would, therefore, be identical to the filament colour. The dehisced anther thus remained at an optimal pollination position.

To determine pollinator preference for different stamen movement patterns, several pollinators (*n* = 30) were observed for each treatment on a sunny day with the visitation rate and visitation duration for each pollinator recorded. We calculated the different visitation rates (total number of visits of 30 pollinators for every flower, for every treatment) and visitation duration (average visitation duration of 30 pollinators for every flower for each treatment) to determine the pollinator preference for the different stamen movement patterns.

### Temporal patterns of pollen dispersal for different stamen batches

To assess the pollen dispersal pattern for different stamen batches, 60 new flowers from *L. cognata* were randomly selected between 10 and 12 August 2015 and bagged before opening to obtain flowers unvisited by pollinators, one flower per individual.

At about 0700 h, when the flowers opened, we unsealed the bags and anthers of the first batch of stamens were collected at 0, 2, 4, 6, 8 and 10 h after flower opening; anthers of the second batch were collected at 24, 26, 28, 30, 32 and 34 h after flower opening. Anthers were placed into 1.5-mL centrifuge tubes, and 1 mL 2 % pectinase was added to the tubes. After mixing for 12 h a 0.1-mL pollen suspension was dropped onto a microscope slide marked with 100 small grids. Pollen numbers in 50 grids were counted and the number of pollen grains for the corresponding period was calculated:

*Remaining pollen grains of corresponding period = pollen grains count in the suspension × 2×10*

### Effects of vertical stamen movement on pollen dispensing

To assess the effects of vertical stamen movement (when anthers move out of flower in two batches) in prolonging the presentation of pollen grains, we carried out a manipulation experiment on 20 randomly selected flowers in the field (10 assigned as controls, and 10 for treated) on 13 August 2015, one flower per individual. For the manipulation treatment, to simulate as if two batches of stamens were moving out of the flower together, we unsealed the bags at 24 h after the flowers opened and fixed the first batch of stamens to the second batch at the top of filaments with a white thread. For the control treatment, we simply bagged the flowers before they opened. Anthers from the first and second batch of stamens were collected at 34 h after the flowers opened, and placed into 1.5-mL centrifuge tubes before the number of pollen grains quantified as described above.

### Effects of horizontal stamen movement on pollen dispensing

To assess the effects of horizontal stamen movement (when the first batch of anthers gets in the way of disseminating pollen from the second of anthers), between 14 and 16 August 2015, 60 flowers (30 assigned as controls and 30 for treated) were randomly selected, bagged, and marked before opening to obtain unvisited flowers, one flower per individual. For the manipulation treatment, we tied stamens to prevent horizontal movement at 24 h after the flowers opened. We unsealed the bags within an hour of flower opening and exposed the flowers to pollinators; each anther was collected after one visitation. Anthers from the second batch of stamens in the two groups were collected after one visitation, and placed in 1.5-mL centrifuge tubes, and the number of pollen grains was counted as described above.

### Effects of number of visits on fruit production

Between 17 and 19 August 2015, 40 buds were randomly selected, bagged, and marked to obtain unvisited flowers, 1 flower per individual. We unsealed the bags 72 h after the flowers opened, and rebagged the flowers after they had been visited 1, 2, 3 or 4 times by pollinators. After the seeds matured in late August, we calculated the fruit set and took capsules back to the lab to record capsule length, diameter, seed number and mean seed mass.

### Effects of horizontal stamen movement on fruit production

From 21 August to 18 September 2015, 60 flowers (30 assigned as controls and 30 for treated) were selected randomly, one flower per individual. For the manipulation treatment, we prevented the horizontal movement of the stamens before the flowers were exposed to pollinators. We calculated the fruit set and took the capsules back to the laboratory to record capsule length, diameter, seed number and mean seed mass in late August after the fruits matured.

### Statistical analyses

All statistical analyses were carried out using SPSS 22.0 for Windows (SPSS Inc., USA). We used two-way ANOVA to assess the differences in stamen length, pollen viability, filament angle, and pollen dispensed across time and collection batches. Two-way ANOVA was also used to test for the effect of time and flowering stage on insect visitation rate and duration. One-way ANOVA was used to test for differences in pistil length and stigma viability at different time after flowering. One-way ANOVA was used to test for differences in fruit set, capsule length, capsule diameter, seed number and mean seed mass at different number of visits. Post hoc multiple comparisons were made using the Bonferroni method after ANOVA tests. An independent samples *t*-test was used to identify differences in fruit set, capsule length, capsule diameter, seed number, mean seed mass, pollen removal, visitation rate and visitation duration between the manipulation and control treatments. Differences were considered significant if *P* < 0.05. Statistical data are expressed as means ± standard errors.

## Results

### Vertical and horizontal movements of stamens and pollen dispersal

**Figure 3.**
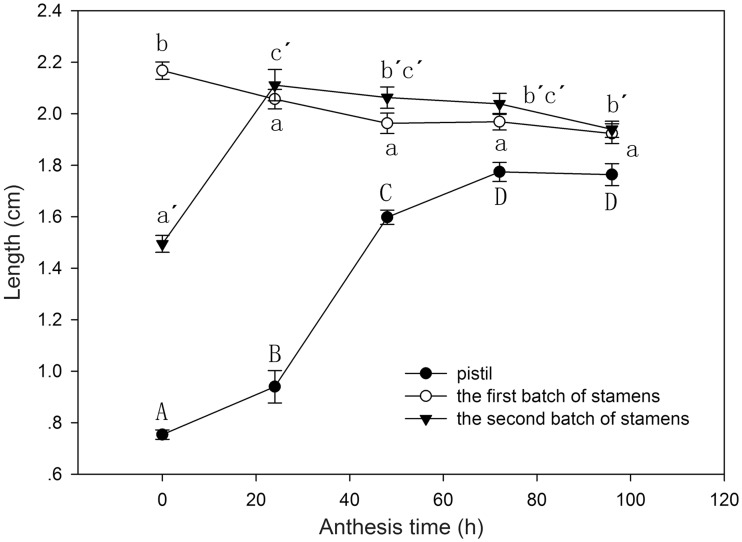
Temporal variation in the lengths of the first and second batches of stamens and pistils during *L. cognata* anthesis. The letters a and b indicate comparisons within the first batch of stamens; a′, b′ and c′ show post hoc comparisons within the second batch of stamens; A, B, C and D are used for post hoc comparisons within pistils.

Two successive vertical stamen movements were observed in *L. cognata* flowers. Flowers opened between 0700 and 0800 h. Five flat petals were located in the same horizontal plane when flowers were fully open (Fig. 1A). The first batch of stamens aggregated at the centre of the flower, which is the optimal pollination position as pollinators place their proboscis into the tubular corolla and come into contact with the stamens or pistils at the centre of the flower. The second batch of stamens was below the cylinder port (Fig. 3). At 30 min after flower opening, pollen grains were released through a longitudinal split in the anthers. We classified these first day flowers as first stage males (M1). The second batch of stamens began to elongate their filaments and slowly raised their anthers above the flower, taking ∼24 h. On the following morning, the second batch of stamens had moved out of the tubular corolla (Figs 1B and 3). We classified these second day flowers as second stage males (M2). Two-way ANOVA suggested that time (*F*_4,__ __190_ = 11.694, *P* < 0.001), stamen batch (*F*_1,__ __190_ = 11.921, *P* = 0.001), and the interaction of these two variables (*F*_4__, __190_ = 34.521, *P* < 0.001) had significant effects on stamen length. It took ∼2 days for the flowers to complete their vertical stamen movements. The female stage (F) began on the third day, at 0800 h, when five pistils were uplifted. At this initial stage, the pistils did not protrude from the tubular corolla (Fig. 3) but were on the flower plane. Gradually, the pistils extended and reached their maximum height on the morning of the fourth day (Figs 1C and 3), and were able to accept pollen. The female stage usually lasted for 3–5 days depending on weather conditions; heavy rain or strong sun light shortened the length of this stage (Fig. 1D).

**Figure 4.**
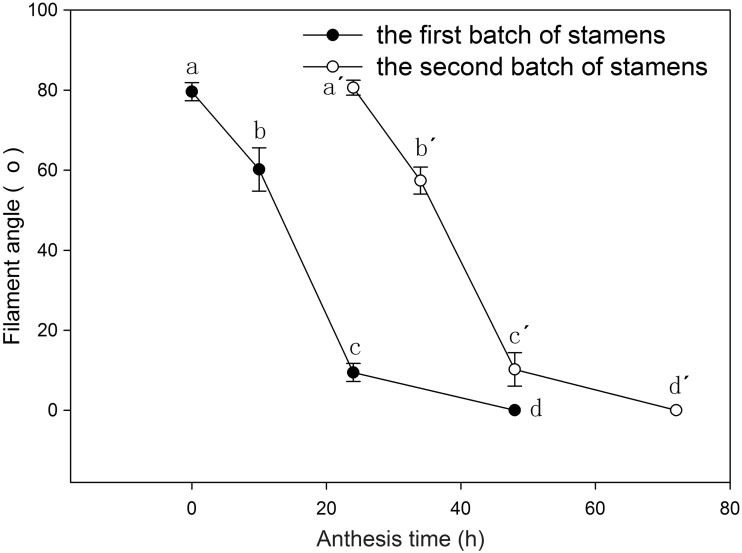
Filament angles of the first and second batches of stamens related to the length of *L. cognata* anthesis. The letters a, b, c and d indicate post hoc comparisons within the first batch of stamens; a′, b′, c′ and d′ show post hoc comparisons within the second batch of stamens.

Horizontal stamen movements were also observed. Immediately after the flowers opened, the first batch of stamens was aggregated at the centre of the flower, and distributed evenly around the tubular corolla (Figs 1A and 4). The stamens then developed curvature, resulting in a decreased angle. The filaments tilted towards the edge of the tubular corolla on the following morning (Fig. 4), whereas the second batch of stamens occupied the optimal pollination position where the first batch of stamens had been located the previous day (Figs 1B and 4). On the third day after flowering, all the first and second batch stamens had moved towards the petals (Fig. 1C). Two-way ANOVA showed that time had a significant effect on the filament angle (*F*_3__, __32_ = 329.136, *P* < 0.001), whereas stamen batch showed no significant effect on filament angle (*F*_1__, __32_ = 0.015, *P* = 0.903) and there was no interaction (F_3__, __32_ = 0.170, *P* = 0.916). It took ∼3 days for the flowers to finish horizontal stamen movement. Interestingly, different optimal positions for the two batches of stamens were available: the first batch of stamens curve at the midline of the petals and the second batch of stamens curve in between the petals.

**Figure 5.**
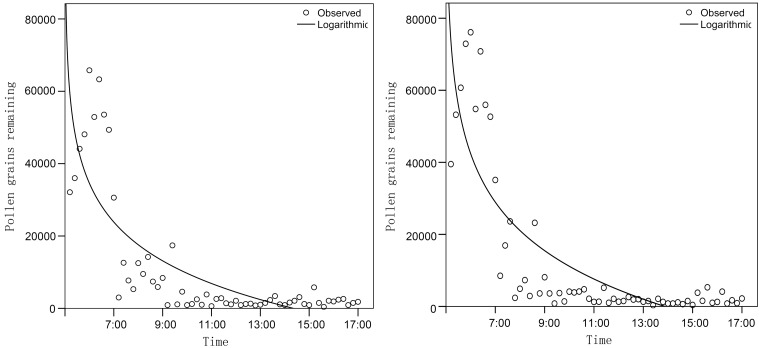
Pollen dispensing from the first and second batches of stamens related to the length of *L. cognata* anthesis.

Anthers of *L. cognata* flowers were pollen-rich, and the pollen content did not show a significant difference between the two stamen batches (*t* = 1.670, *df *=18, *P* = 0.112; Fig. 5). Two-way ANOVA showed that time had a significant effect on pollen dispersal (F_5__, __108_ = 218.901, *P* < 0.001), whereas stamen batch did not have a significant effect (*F*_1__, __108_ = 2.501, *P* = 0.117) and there was no interaction (*F*_5__, __108_ = 2.071, *P* = 0.075), suggesting that pollen dispersal was not affected by stamen batch. Post hoc tests found that the number of pollen grains at 0700 h was significantly higher than 0900 h (*P* < 0.001), 1100 h (*P* < 0.001), 1300 h (*P* < 0.001), 1500 h (*P* < 0.001) and 1700 h (*P* < 0.001); a significantly higher number of pollen grains were present at 0900 h than at 1100 h (*P* = 0.041), 1300 h (*P* = 0.006), 1500 h (*P* = 0.009) and 1700 h (*P* = 0.012). These analyses suggested that dispersal of the pollen dispensed from both the first and second batches of stamens occurred mainly in a period before 1100 h, with 94.8 ± 0.79 % and 92.9 ± 3.39 % pollen grains being shed (Fig. 5). Most pollen grains (95.85 ± 0.69 %, the sum of the two stamen batches) were successfully shed by 10 h after anther dehiscence.

### Viability changes in pollen and stigma

**Figure 6.**
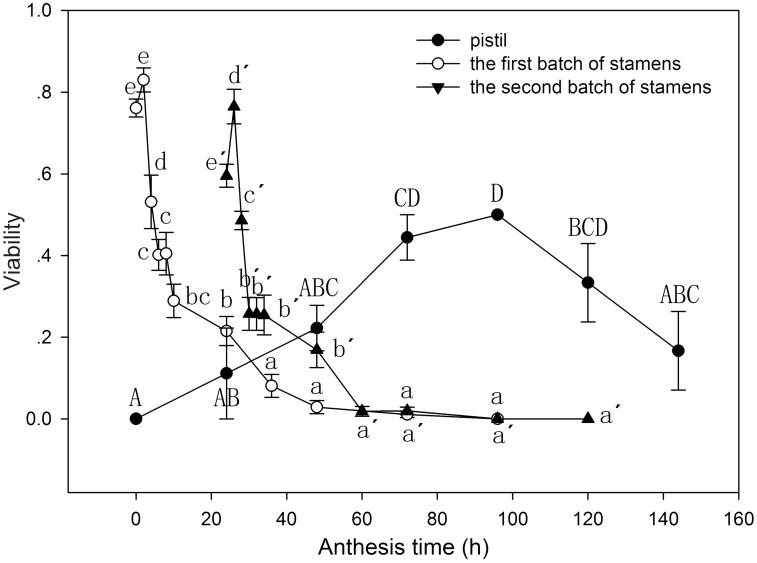
Viability changes in pollen and stigma related to the length of *L. cognata* anthesis. The letters a, b, c, d and e indicate comparisons within the first batch of stamens; a′, b′, c′, d′ and e′ show post hoc comparisons within the second batch of stamens; A, B, C and D are used for post hoc comparisons within pistils.

On the morning of flower opening, the first batch of stamens functioned as pollen donors at the optimal pollination position, with the highest pollen viability of 83.0 ± 2.94 % observed at 0900 h. The viability of the pollen decreased as the filament curved progressively downwards. Moreover, it was observed in the following morning at 8000 h that pollen had lost viability considerably (Fig. 6). When the optimal pollination position was occupied by the second batch of stamens, the highest pollen viability was 76.5 ± 4.23 % at 0900 h (Fig. 6), which declined gradually until the stamens were present on the petals in the evening. Two-way ANOVA suggested that both time (*F*_17__, __418_ = 78.448, *P* < 0.001) and stamen batch (*F*_1__, __418_ = 33.452, *P* < 0.001) had significant effects on pollen viability, and that the interaction between time and stamen batch also had a significant effect on pollen vitality (*F*_3__, __418_ = 15.397, *P* < 0.001).

Stigma viability increased as the stigma moved into the optimal pollination position, and the highest viability occurred on the morning of the fifth day (50.0 ± 0.00 %).

### Pollinator preference for different flowering stages

Our results indicated that *Bhutanitis yulongensis* was the major pollinator of *L. cognata*. It often rested randomly on two petals of the flowers, placing its proboscis into the tubular corolla, and hovered at the flower to drink nectar secreted from the base of the ovary. Because nectar was produced by the disc at the base of the ovary, the pollinator foraged mainly around the centre of the flower, thus touching the dehiscing anthers or stigma located at the centre of the flower more frequently. Numerous pollen grains adhered to the face or other parts of the body of the insect during the visitation; these pollen grains were transported to the pistil of the next flower in the female stage.

**Figure 7.**
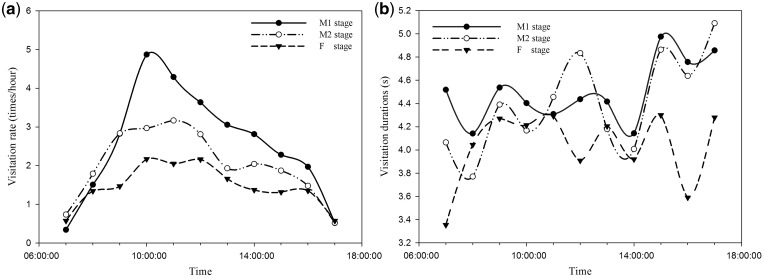
Pollinator' preferences for first day male flowers (M1), second day male flowers (M2), and third day female flowers (F) in *L. cognata.* (A) Pollinator visitation rate (times per hour) for the M1, M2 and F stage flowers after flowering. (B) Pollinator visitation duration (seconds per visitation) for M1, M2 and F stage flowers after flowering.

Two-way ANOVA showed that both time (*F*_2__, __72_ = 26.139, *P* < 0.001) and flower stage (*F*_2__, __72_ = 20.230, *P* < 0.001) had a significant effect on insect visitation rates, but there was no interaction (*F*_22__, __72_ = 1.485, *P* = 0.118). Time (*F*_2__, __5386_  = 4.120, *P* < 0.001) and flower stage (*F*_11__, __5386_ =  9.903, *P* < 0.001) also had a significant effect on insect visitation duration, and there was a significant interaction (*F*_22__, __5386_ = 1.890, *P* = 0.007). The insect visitation rate between 0900 and 1000 h, and the visitation duration between 1600 and 1700 h were higher than that at any other time of the day (Fig. 7A and B). Post hoc tests found pollinators had a significantly higher visitation rate for M1 flowers than M2 flowers (*P* = 0.002) and F flowers (*P* < 0.001); and a significantly longer period of number of visits on M1 flowers (*P* < 0.001) and M2 flowers (*P* = 0.002) than F flowers, suggesting that F flowers were less favoured by pollinators. Nevertheless, the total visitation rate of F stage flowers was still as high as 6.7 ± 0.98 times per hour between 0700 and 1100 h.

### Effects of vertical stamen movement on pollinator preference and pollen dispersal

**Figure 8.**
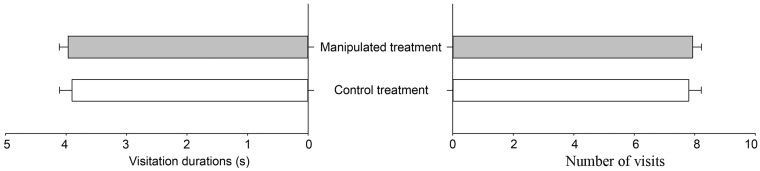
Number of visits and visitation duration of floral visitors of control and manipulated flowers in *L. cognata*.

When the first batch of stamens was fixed to the second batch at the centre of the flower on the morning of the second day, the manipulated flowers had slightly higher visitation rate and visitation duration than control flowers did (Fig. 8). However, an independent samples *t*-test revealed that the vertical stamen movement had no significant effect on visitation rate (*t* = 0.402, *df *= 28, *P* = 0.691) and visitation duration (*t* = −0.325, *df *= 234, *P* = 0.746).

As the first batch of stamens were fixed to the second batch at the centre of the flower to simulate as if they were moving out of the flower together, most pollen grains from both batches of stamens were successfully shed, on average 98.0 ± 1.84 % and 97.8 % (1.86 %) of the pollen grains were removed in the first batch and second batch, 10 h after exposure to pollinators. This suggests that almost all pollen grains were dispersed within 1 day after flowering when vertical stamen movement was inhibited.

### Effects of horizontal stamen movement on pollen dispersal

**Figure 9.**
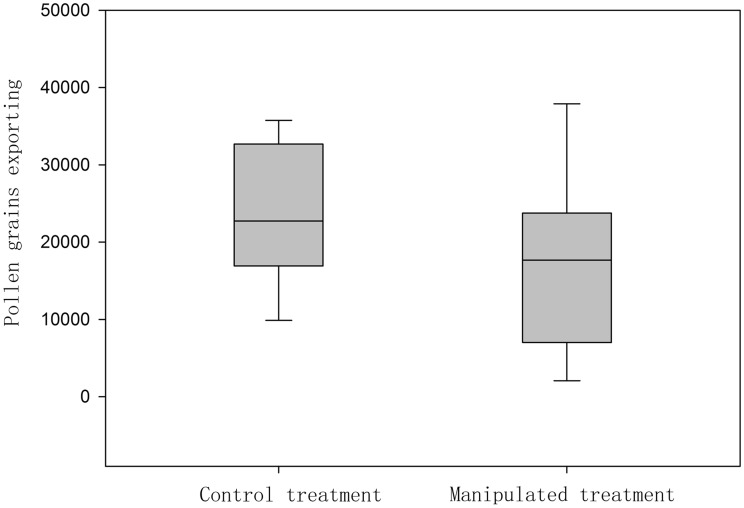
Number of pollen grains dispensed at visitation in control and manipulated flowers in *L. cognata*.

The number of pollen grains dispensed from the control and manipulated flowers in one visitation were 23 903 ± 1795 and 17 877 ± 2226, respectively (Fig. 9). This suggested that pollen dispersal declined by 25.21 ± 1.80 % in one visitation when horizontal stamen movement was inhibited; this decrease was statistically significant (*t* = 2.107, *df *= 18, *P* = 0.039).

### Effects of number of visits on fruit production

Fruit set increased significantly with number of pollinator visits (*F*_3__, __8_ = 10.19; *P* = 0.004; Table 1). One visitation during 0700–1100 h resulted in an average fruit set of 22.20 ± 14.70 %; however, capsule length (*F*_1__, __18_ = 16.043; *P* = 0.001), capsule diameter (*F*_1__, __18_ = 5.201; *P* = 0.035), seed number (*F*_1__, __18_ = 47.246; *P* < 0.001), and mean seed mass (*F*_1__, __18_ = 19.406; *P* < 0.001) were significantly lower than those under natural conditions when pollinators were allowed to freely visit the flowers. Three visitations by pollinators caused a higher fruit set (77.78 ± 5.56 %) than one visitation, with no significant differences in capsule length (*F*_1__, __18_ = 0.003, *P* = 0.960), capsule diameter (*F*_1__, __18_ = 1.537, *P* = 0.231), seed number (*F*_1__, __18_ = 1.017, *P* = 0.327) and mean seed mass (*F*_1__, __18_ = 0.013, *P* = 0.910) compared with fruits formed by two visitations or more. These data indicate that two pollinator visitations during 0700–1100 h were sufficient for effective fruit production in *L. cognata*.

**Table 1.** Summary of fruit set, capsule length, capsule diameter, seed number, mean seed mass of *Lychnis cognata* under different number of visits. Note: ‘n’ means number of flowers ‘a’ and ‘b’ indicate significant differences.

Number of visits	Fruit set (%)	Capsule length (cm)	Capsule diameter (cm)	Seed number	Mean seed mass (g)
One (n = 10)	22.2±14.70a	1.07±0.085a	0.66±0.017a	22.8±0.28a	0.28±0.04a
Two (n = 10)	50.0±0.00b	1.47±0.050b	0.72±0.016a	69.2±0.61b	0.81±0.11b
Three (n = 10)	77.8±5.56b	1.47±0.059b	0.68±0.025a	85.3±1.47b	0.79±0.14b
Four (n = 10)	77.8±5.56b	1.48±0.063b	0.67±0.029a	71.7±0.90b	0.89±0.10b

### Effects of horizontal stamen movement on fruit production

An independent samples *t*-test revealed that horizontal stamen movement had a significant effect on fruit set (*t* = −6.327, *df* = 58, *P* = 0.003; Table 2). Fruit set decreased dramatically when horizontal stamen movement was inhibited. The capsule length (*t* = 3.800, *df* = 58, *P* < 0.001), seed number (*t* = 3.811, *df* = 58, *P* < 0.001), and mean seed mass (*t* = 3.992, *df* = 58, *P* < 0.001) of manipulated flowers were significantly lower than those of fruits formed under natural conditions. In contrast, capsule diameter was not significantly affected (*t* = 1.332, *df* = 58, *P* = 0.188).

**Table 2.** Summary of fruit set, capsule length, capsule diameter, seed number, mean seed mass of *Lychnis cognata* under different stamen movement pattern.

Treatment	Fruit set (%)	Capsule length (cm)	Capsule diameter (cm)	Seed number	Mean seed mass (g)
Control flowers	77.8±5.56	1.50±0.033	0.65±0.015	70.4±4.68	0.86±0.059
Manipulative flowers	34.4±4.01	1.30±0.041	0.62±0.019	46.9±4.00	0.52±0.059

## Discussion

This study provides the first evidence that slow stamen movement in *L. cognata* reflects an adaptation to present pollen gradually to pollinators and, more importantly, decreases concurrent male**–**male and male**–**female sexual interference in flowers. This behaviour may promote pollen export and reception. These results demonstrate that the pressure to decrease concurrent male**–**male and male**–**female sexual interference should be recognized as an important selective force for slow stamen movement.

### The process of slow stamen movement

Slow stamen movement in *L. cognata* could be resolved into vertical and horizontal movements: (i) in vertical movement, the 10 stamens in one flower elongated their filaments in two batches and anthers dehisced when they moved out of the flower; (ii) in horizontal movement, anther-dehisced stamens bent out of the floral centre before the next stamens or pistils moved out of the flower. The pollen or stigma located in the flower centre (referred to as the optimal pollination position) displayed high viability (83.0 ± 2.94 % for M1 pollen, 76.5 ± 4.23 % for M2 pollen, 50.0 ± 0.0 % for F stigma). Under natural conditions, pollen viability often decreases quickly after anther dehiscence (Liu *et al.* 2011). However, in *L. cognata*, the viability of M1 pollen decreased more slowly than that of M2 pollen; only 78.5 ± 3.55 % of M1 pollen lost viability compared to 83.1 ± 4.32 % of M2 at 24 h after anther dehiscence. Furthermore, stigma viability increased gradually to reach a maximum level on the third day after flowering.

We propose that the changes in pollen and stigma viability reflect an adaption to prevent self-fertilization. Even if some self-pollen is transferred to the stigma, pollen has low viability when the stigma is receptive, and the stigma has low receptivity when pollen is viable, effectively preventing self-fertilization, as is the case in dichogamy (Lloyd and Webb 1986).

We also found that pollinators had a significant preference for M1 flowers, with the visitation rate averaging 28.7 ± 2.00 times per day and visitation duration averaging 4.5 ± 0.48 s, compared with that for M2 and F flowers. A similar phenomenon was also described in *Polygala vayredae*, in which the floral lifespan of open-pollinated flowers was negatively correlated with pollinator visitation rates (Castro and Silveira 2008). This is probably related to the degree of bright coloration of the flowers (Lázaro *et al.* 2013); fresh flowers are more favoured by pollinators, and, therefore, pollinators prefer male first stage flowers. In addition, it is possible that this is related to the nectar rhythm of the flowers (Sánchez-Lafuente *et al.* 2005), which gradually decreases after flowering.

### Temporally separated pollen dispersal explains the stamen vertical movement

Though visitation rate (*t* = 0.319, *P* = 0.761) and visitation duration (*t* = −0.435, *P* = 0.669) were not affected when stamen vertical movement was stopped, total visitation time was doubled when stamen vertical movement occurred, because if there are two sets of stigmas, they are available for two times as much time. Meanwhile, manipulation experiments revealed that almost all pollen grains were dispersed in 1 day when stamen vertical movement was stopped, and pollen grains took 2 days to disperse when stamen vertical movement occurred. This confirmed the ‘pollen presentation theory’ that vertical stamen movements function to present the pollen to a greater number of pollinators (Percival 1955; Fenster *et al.* 2009). Armbruster (2014) also proposed granular pollen results in low precision in pollination. If pollen deposition on the stigma is imprecise, it is better if the pollinator does not remove all of it at one time. Thomson *et al.* (2000) found that plants that receive more pollinator visits can maximise the amount of pollen they donate to stigmas by presenting their pollen in many small doses rather than by presenting all of it at once. On average, a bumblebee deposits 0.6 % of the pollen from *Erythronium grandiflorum* flowers onto the stigmas of subsequently visited flowers (Harder and Thomson 1989). Restriction in pollen removal and deposition could be achieved by vertical stamen movement that presents only half of the pollen at one time; this would limit the amount of pollen that any given pollinator could remove during a single visit.

The apparent reproductive advantage of stamen movement raises the question of the inclusion of five stamens in each batch, instead of one or two. *Lychnis cognata* has actinomorphic flowers consisting of five petals, which are generally populated by ecologically generalized pollinators that are not specific in their pollination (Lazaro and Totland 2014). Pollinators approach the flower from any direction, often land on two of the five petals, randomly. The inclusion of five stamens simultaneously in each batch movement would increase the probability of successful pollen removal by pollinators from each direction. Furthermore, pollinators can have a strong preference for floral symmetry (Jabbour *et al.* 2008). Therefore, floral structure in *L. cognata* may be an adaptation to the increased preference for floral symmetry of pollinators, and thus increases the probability of successful pollen removal by pollinators from any direction.

Another interesting question is why the filament elongation and formation of curvature takes nearly a whole day to reach the maxima? We speculate that the rates of movement may be an adaptation to pollen longevity, pollinator behaviour, and weather conditions. *Lychnis cognata* has relatively short pollen longevity; the viability declines quickly after flowering and it is completely lost after 24 h of flowering. Therefore, presenting a new batch of pollen with high viability to replace the first batch of pollen could be a strategy to prolong effective pollen dispersal. Huang *et al.* (2002) suggested that movement of the flower stalk could maintain pollen viability in a rainy habitat with a scarcity of pollinators. Similarly, stamen movement is modulated by abiotic (light and temperature) as well as biotic stimuli (pollinator availability and visitation frequency) (Henning and Weigend 2013). As pollinators are abundant in our study area, *L. cognata* exports almost all of its pollen within 10 h (1700 h) of anther dehiscence under high visitation. Pollinators visit flowers frequently in the morning with only a few visits after 1700 h. As a result, uplifting the second batch of stamens the next morning after flowering not only efficiently replaces the first batch of pollen, but also avoids reduction in pollen viability due to low night temperatures (Shaked *et al.* 2004) and the adverse effect of dew in the early morning (Ye and Peng 2011).

M2 flowers received a mean of 6.65 ± 0.98 visits during the 0700–1100 h period. One visitation during this period produced ∼22.2 ± 14.70 % fruit set, although with low capsule length, capsule diameter, seed number, and mean seed mass. After three visitations during the same period, there was no significant difference in these fruit values, indicating that three visits were enough for female reproductive success. Obviously, the presentation of all the pollen at once and the visitation rate is sufficient for fruiting. This high efficiency of pollination has also been observed in many other plant species (Vaughton and Ramsey 2008). In *Melampyrum roseum* var. japonicum flowers, three or four visits from pollinators did not further increase seed set and pollen removal (Hiei and Suzuki 2001).

### Interaction between male–male and male–female sexual interference explains stamen horizontal movement

The presence of a large number of stamens in a small flower can cause interference among individual stamens and has been recognized as an important selective force in floral evolution (Ren and Bu 2014). Manipulation experiments showed that when the first batch of stamens was fixed at the centre of the flower and arranged evenly alongside the second batch of stamens and around the tubular corolla, pollinators came into contact with the second batch of stamens less frequently. As a result, pollen dispersal from one pollinator visitation decreased by 25.21 ± 1.80 %. These results demonstrated that stamen horizontal movement in *L. cognata* reflects an adaptation to decrease the interference between dehisced and dehiscing anthers. Similarly, observed in *Parnassia palustris*, the dehisced anthers blocked the access of a dehiscing anther to pollinators, resulting in a nearly one-third decrease in contact frequency and pollen removal from dehiscing anthers (Ren and Bu 2014). The spatial separation of anthers by stamen movement is similar to tetradynamous stamens (Conner *et al.* 2003; Kudo 2003) and didynamous stamens (Ren and Tang 2010). However, research on male**–**male interference has received attention only recently, such as in Asclepiadaceae (Cocucci *et al.* 2014) and Saxifragaceae (Ren and Tang 2012, Ren and Bu 2014).

Floral hermaphroditism results in conflict and compromise in the parental roles of plants during pollination and mating, which might produce sexual interference between maternal and paternal functions, resulting in gamete wastage and reduced fitness (Barrett 2002). Stamen horizontal movement could cause herkogamy, which is usually interpreted as a mechanism to reduce sexual interference, in that it increases outcrossing and decreases self-fertilization (Webb and Lloyd 1986; Zhang 2004). Since Darwin’s observation of the reconfiguration of pollinia in orchids, which he concluded was a means of reducing self-fertilization, diverse floral movements have been investigated and various hypotheses have been proposed to explain their adaptive significance (Ruan and Silva 2011). This study further revealed that when the first and second batch of stamens were fixed at the centre of the flower and placed evenly alongside the pistils around the tubular corolla, they interfered with pollen reception, resulting in a decrease in seed set and capsule length, capsule diameter, seed number, and mean seed mass. Thus, stamen horizontal movement in *L. cognata* could be interpreted as an adaptation capable of decreasing male**–**female sexual interference. In other species, such as *Parnassia* species (Ren and Tang 2012; Armbruster *et al.* 2014) and members of Rubiaceae (Hua *et al.* 2012), staggered pollen and stigma presentation with spatial correspondence can reduce male**–**female interference.

## Conclusions

The results in this study suggested that slow stamen movement in *L. cognata* presents pollen gradually to pollinators and, moreover, reflects an adaptation capable of decreasing concurrent male**–**male and male**–**female interference. This study provided insight into the selective forces in the evolution of floral traits. Future studies will focus on the mechanism of slow stamen movement in *L. cognata*.

## Sources of Funding

This study was funded by the National Natural Science Foundation of China (Grant No. 31470446) and the open fund of the State Environmental Protection Key Laboratory of Wetland Ecology and Vegetation Restoration, Northeast Normal University. 

## Contributions by the Authors

L.-Y.W., Z.-H.T. and L.-X.S. designed research; L.-Y.W. and Y.B. performed research; L.-Y.W., Z.-H.T., P.W. and H.-X.W. analysed data; and L.-Y.W., Z.-H.T., C.-G.H. and H.-X.W. wrote the paper.

## Conflicts of Interest Statement

None declared.

## References

[plx018-B1] Armbruster WS. 2014 Floral specialization and angiosperm diversity: phenotypic divergence, fitness trade-offs and realized pollination accuracy. AoB Plants 6:1–24. 10.1093/aobpla/plu003 PMC4038416 24790124

[plx018-B2] Armbruster WS, Corbet SA, Vey AJM, Liu SJ, Huang SQ. 2014 In the right place at the right time: *Parnassia* resolves the herkogamy dilemma by accurate repositioning of stamens and stigmas. Annals of Botany 113:97–103. 24265349 10.1093/aob/mct261 PMC3864732

[plx018-B3] Barrett SCH. 2002 Sexual interference of the floral kind. Heredity 88:154–159. 11932774 10.1038/sj.hdy.6800020

[plx018-B4] Castro S, Silveira PL. 2008 Effect of pollination on floral longevity and costs of delaying fertilization in the out-crossing *Polygala vayredae Costa* (Polygalaceae). Annals of Botany 102:1043–1048. 18829587 10.1093/aob/mcn184 PMC2712401

[plx018-B5] Cocucci AA, Salvador M, Matías B, Wiemer AP, Alicia S. 2014 The buck in the milkweed: evidence of male**–**male interference among pollinaria on pollinators. New Phytologist 203:280–286. 24645822 10.1111/nph.12766

[plx018-B6] Conner JK, Rice AM, Stewart C, Morgan MT. 2003 Patterns and mechanisms of selection on a family-diagnostic trait: evidence from experimental manipulation and lifetime fitness selection gradients. Evolution 57:480–486. 12703937 10.1111/j.0014-3820.2003.tb01539.x

[plx018-B7] Cota-Sánchez JH, Almeida OJ, Falconer DJ, Choi HJ, Bevan L. 2013 Intriguing thigmonastic (sensitive) stamens in the *Plains Prickly Pear Opuntia polyacantha* (Cactaceae). Flora-Morphology, Distribution, Functional Ecology of Plants 208:381–389.

[plx018-B8] Du W, Qin KZ, Wang XF. 2012 The mechanism of stamen movement in *Chimonanthus praecox* (Calycanthaceae): differential cell growth rates on the adaxial and abaxial surfaces of filaments after flower opening. Plant Systematics & Evolution 298:561–567.

[plx018-B9] Fenster CB, Armbruster WS, Dudash MR. 2009 Specialization of flowers: is floral orientation an overlooked first step? New Phytologist 183:502–506. 19422542 10.1111/j.1469-8137.2009.02852.x

[plx018-B10] Harder LD, Thomson JD. 1989 Evolutionary options for maximizing pollen dispersal of animal-pollinated plants. The American Naturalist 133:323–344.

[plx018-B11] Henning T, Weigend M. 2013 Beautiful, complicated–and intelligent? Novel aspects of the thigmonastic stamen movement in Loasaceae. Plant Signaling & Behaviour 8:1–5. 10.4161/psb.24605 PMC3909056 23603953

[plx018-B12] Hiei K, Suzuki K. 2001 Visitation frequency of *Melampyrum roseum var. japonicum* (Scrophulariaceae) by three bumblebee species and its relation to pollination efficiency. Canadian Journal of Botany 79:1167–1174.

[plx018-B13] Hua L, Fan XL, Xiang Z, Gao JY. 2012 Self-interference is reduced in a secondary pollen presentation species, *Duperrea pavettifolia* (Rubiaceae). Flora - Morphology, Distribution, Functional Ecology of Plants 207:895–902.

[plx018-B14] Huang SQ, Takahashi Y, Dafni A. 2002 Why does the flower stalk of *Pulsatilla cernua* (Ranunculaceae) bend during anthesis? American Journal of Botany 89:1599–1603. 21665586 10.3732/ajb.89.10.1599

[plx018-B15] Jabbour F, Damerval C, Nadot S. 2008 Evolutionary trends in the flowers of Asteridae: is polyandry an alternative to zygomorphy? Annals of Botany 102:153–165. 18511411 10.1093/aob/mcn082 PMC2712368

[plx018-B16] Joan E, Dwight W, Sarah K, Laskowski MJ. 2005 Botany: a record-breaking pollen catapult. Nature 435:164. 15889081 10.1038/435164a

[plx018-B17] Kudo G. 2003 Anther arrangement influences pollen deposition and removal in hermaphrodite flowers. Functional Ecology 17:349–355.

[plx018-B18] Lázaro A, Jakobsson A, Totland Ø. 2013 How do pollinator visitation rate and seed set relate to species’ floral traits and community context? Oecologia 173:881–893. 23579571 10.1007/s00442-013-2652-5

[plx018-B19] Lanteri S, Acquadro A, Comino C, Mauro R, Mauromicale G, Portis E. 2006 A first linkage map of globe artichoke (*Cynara cardunculus* var. *scolymus* L.) based on AFLP, S-SAP, M-AFLP and microsatellite markers. TAG. Theoretical & Applied Genetics. Theoretische Und Angewandte Genetik 112:1532–1542. 16565844 10.1007/s00122-006-0256-8

[plx018-B20] Lazaro A, Totland O. 2014 The influence of floral symmetry, dependence on pollinators and pollination generalization on flower size variation. Annals of Botany 114:157–165. 24838838 10.1093/aob/mcu083 PMC4071101

[plx018-B21] Li P, Zheng GL, Dafni A, Luo YB. 2010 Reproductive biology of an alpine orchid *Phaius delavayi*. Plant Systematics and Evolution 286:167–173.

[plx018-B22] Liu H, Gao Y, Dong NG, Xu HZ, Pei D. 2011 Study on pollination activity of pistillate and staminate flowers in walnut excellent varieties. Journal of Beijing Forestry University 33:119–123.

[plx018-B23] Liu KW, Liu ZJ, Huang LQ, Li LQ, Chen LJ, Tang GD. 2006 Pollination: self-fertilization strategy in an orchid. Nature 441:945–946. 16791185 10.1038/441945a

[plx018-B24] Lloyd DG, Webb CJ. 1986 The avoidance of interference between the presentation of pollen and stigmas in angiosperms I. Dichogamy. New Zealand Journal of Botany 24:135–162.

[plx018-B25] Percival MS. 1955 The presentation of pollen in certain angiosperms and its collection by *Apis Mellifera*. New Phytologist 54:353–368.

[plx018-B26] Ren MX. 2010 Stamen movements in hermaphroditic flowers: diversity and adaptive significance. Chinese Journal of Plant Ecology 34:867–875.

[plx018-B27] Ren MX, Bu ZJ. 2014 Is there ‘anther-anther interference’ within a flower? Evidences from one-by-one stamen movement in an insect-pollinated plant. PLoS One 9:1–7. 10.1371/journal.pone.0086581 PMC3903572 24475150

[plx018-B28] Ren MX, Tang JY. 2010 Anther fusion enhances pollen removal in *Campsis grandiflora*, a hermaphroditic flower with didynamous stamens. International Journal of Plant Sciences 171:275–282.

[plx018-B29] Ren MX, Tang JY. 2012 Up and down: stamen movements in *Ruta graveolens* (Rutaceae) enhance both outcrossing and delayed selfing. Annals of Botany 110:1017–1025. 22875813 10.1093/aob/mcs181 PMC3448434

[plx018-B30] Ruan CJ, Silva JATd. 2011 Adaptive Significance of Floral Movement. Critical Reviews in Plant Sciences 30:293–328.

[plx018-B31] Sánchez-Lafuente AM, Guitián J, Medrano M, Herrera CM, Rey PJ, Cerdá X. 2005 Plant traits, environmental factors, and pollinator visitation in winter-flowering *Helleborus foetidus* (Ranunculaceae). Annals of Botany 96:845–852. 16093269 10.1093/aob/mci236 PMC4247050

[plx018-B32] Shaked R, Rosenfeld K, Pressman E. 2004 The effect of low night temperatures on carbohydrates metabolism in developing pollen grains of pepper in relation to their number and functioning. Scientia Horticulturae 102:29–36.

[plx018-B33] Thomson JD, Wilson P, Valenzuela M, Malzone M. 2000 Pollen presentation and pollination syndromes, with special reference to *Penstemon*. Plant Species Biology 15:11–29.

[plx018-B34] Vaughton G, Ramsey M. 2008 Floral display, pollinator visitation and reproductive success in the dioecious perennial herb *Wurmbea dioica* (Liliaceae). Muscle & Nerve 37:403–405. 28308473 10.1007/s004420050495

[plx018-B35] Wang LY, Wang HX, He CG, Sheng LX, Tang ZH. 2017 An irreversible division of labor through a sexually dependent system in the clonal plant *Iris laevigata* (Iridaceae). Ecosphere 8:e01757.

[plx018-B36] Webb CJ, Lloyd DG. 1986 The avoidance of interference between the presentation of pollen and stigmas in angiosperms II. Herkogamy. New Zealand Journal of Botany 24:163–178.

[plx018-B37] Worboys JS, Jackes RB. 2005 Pollination processes in *Idiospermum australiense* (Calycanthaceae), an arborescent basal angiosperm of Australia’s Tropical Rain Forests. Plant Systematics and Evolution 251:107–117.

[plx018-B38] Ye YH, Peng SL. 2011 Review of dew action effect on plants. Acta Ecologica Sinica 31:3190–3196.

[plx018-B39] Zhang DY. 2004 Plant life-history evolution and reproductive ecology. Beijing: Science Press.

